# Initial validation of the Argentinean Spanish version of the PedsQL™ 4.0 Generic Core Scales in children and adolescents with chronic diseases: acceptability and comprehensibility in low-income settings

**DOI:** 10.1186/1477-7525-6-59

**Published:** 2008-08-07

**Authors:** Mariana Roizen, Susana Rodríguez, Gabriela Bauer, Gabriela Medin, Silvina Bevilacqua, James W Varni, Veronica Dussel

**Affiliations:** 1 Committee on Quality of Life, Hospital de Pediatria Prof. Dr. Juan P Garrahan, Pichincha 1890, Buenos Aires, (1414), Argentina; 2 Department of Research, Hospital de Pediatria Prof. Dr. Juan P Garrahan, Buenos Aires, Argentina; 3 Department of Neonatology, Hospital de Pediatria Prof. Dr. Juan P Garrahan, Buenos Aires, Argentina; 4 Department of Pulmonology, Hospital de Pediatria Prof. Dr. Juan P Garrahan, Buenos Aires, Argentina; 5 Hospital Marañon, Madrid, Spain; 6 Palliative Care Team, Hospital de Pediatria Prof. Dr. Juan P Garrahan, Buenos Aires, Argentina; 7 Department of Pediatrics, College of Medicine, Texas A & M University, College Station, Texas, USA; 8 Department of Landscape Architecture and Urban Planning, College of Architecture, Texas A & M University, College Station, Texas, USA; 9 Center for Outcomes and Policy Research and Department of Pediatric Oncology, Dana-Farber Cancer Institute, 44 Binney St (SM-215), Boston, 02115, MA., USA; 10 Department of Hematology/Oncology, Children's Hospital, Boston, 02115, MA, USA

## Abstract

**Background:**

To validate the Argentinean Spanish version of the PedsQL™ 4.0 Generic Core Scales in Argentinean children and adolescents with chronic conditions and to assess the impact of socio-demographic characteristics on the instrument's comprehensibility and acceptability. Reliability, and known-groups, and convergent validity were tested.

**Methods:**

Consecutive sample of 287 children with chronic conditions and 105 healthy children, ages 2–18, and their parents. Chronically ill children were: (1) attending outpatient clinics and (2) had one of the following diagnoses: stem cell transplant, chronic obstructive pulmonary disease, HIV/AIDS, cancer, end stage renal disease, complex congenital cardiopathy. Patients and adult proxies completed the PedsQL™ 4.0 and an overall health status assessment. Physicians were asked to rate degree of health status impairment.

**Results:**

The PedsQL™ 4.0 was feasible (only 9 children, all 5 to 7 year-olds, could not complete the instrument), easy to administer, completed without, or with minimal, help by most children and parents, and required a brief administration time (average 5–6 minutes). People living below the poverty line and/or low literacy needed more help to complete the instrument. Cronbach Alpha's internal consistency values for the total and subscale scores exceeded 0.70 for self-reports of children over 8 years-old and parent-reports of children over 5 years of age. Reliability of proxy-reports of 2–4 year-olds was low but improved when school items were excluded. Internal consistency for 5–7 year-olds was low (α range = 0.28–0.76). Construct validity was good. Child self-report and parent proxy-report PedsQL™ 4.0 scores were moderately but significantly correlated (ρ = 0.39, p < 0.0001) and both significantly correlated with physician's assessment of health impairment and with child self-reported overall health status. The PedsQL™ 4.0 discriminated between healthy and chronically ill children (72.72 and 66.87, for healthy and ill children, respectively, p = 0.01), between different chronic health conditions, and children from lower socioeconomic status.

**Conclusion:**

Results suggest that the Argentinean Spanish PedsQL™ 4.0 is suitable for research purposes in the public health setting for children over 8 years old and parents of children over 5 years old. People with low income and low literacy need help to complete the instrument. Steps to expand the use of the Argentinean Spanish PedsQL™ 4.0 include an alternative approach to scoring for the 2–4 year-olds, further understanding of how to increase reliability for the 5–7 year-olds self-report, and confirmation of other aspects of validity.

## Background

The shift to family/patient-centered models of care has increased the need for patient reported outcomes. Valid and reliable health-related quality of life (HRQOL) instruments are therefore expected to be in the armamentarium of clinicians and health service researchers [1,2].

The only HRQOL instrument that has been validated in Argentinean children is the Child's Health Questionnaire (CHQ) in children with Juvenile Rheumatoid Arthritis [3,4]. One of the limitations of this instrument however, is that it does not include the child's perspective for children younger than 10 years of age.

The Pediatric Quality of Life Inventory™ (PedsQL™) 4.0 Generic Core Scales is a generic HRQOL instrument for children and adolescents, originally developed by Varni et al. in U.S. English and U.S Spanish [5]. It measures four domains (physical, emotional, social, and school functioning) and has age and respondent specific versions for child self-report ages 5–18 and parent proxy-report for ages 2–18. The PedsQL™ has shown good internal consistency (α = 0.88 child, and α = 0.90 parent report)[6,7] and has been widely used for group comparisons. The construct validity of PedsQL is supported by results from large samples of children from the US [7-10]and several other countries [11-16] where the instrument has been translated using accepted cross cultural language adaptation methods[17]. These studies have given support to the instrument's ability to discriminate between healthy children and those with chronic conditions[7,11,12,15,16,18] and among different chronic conditions[16,19-21]. Responsiveness, i.e. score change after an intervention, has been reported for specific conditions such as rheumatic diseases[22], headaches[23], and cancer[24,25] and sensitivity, i.e. ability to distinguish among severity groups, for heart disease[7], obesity[21] and cancer[24,25] has also been described. In addition, the PedsQL is able to discriminate among children from lower socioeconomic strata[8,11] and predict variation in health care utilization and costs[26,27].

The aim of this study was to validate the Argentinean Spanish version of the PedsQL™ 4.0 in children and adolescents with chronic conditions. Given that families who receive care at public health settings in Argentina come from low income sectors, usually have low literacy skills, and are not used to self-reporting their health status, we specially focused on the impact of socio-demographic characteristics on overall comprehensibility and acceptability.

## Methods

### Subjects

Patients were considered eligible if they were: (1) 2–18 years old, (2) receiving outpatient care at Hospital Nacional de Pediatria Juan P Garrahan, and (3) had one of the following conditions: Allogeneic Hematopoietic Stem Cell Transplantation (SCT), Chronic Obstructive Pulmonary Disease requiring domiciliary oxygen (COPD), Human Immunodeficiency Virus infection or Acquired Immune Deficiency Syndrome (HIV/AIDS), Cancer, End Stage Renal Disease (ESRD) requiring dialysis or transplant, or a Complex Congenital Cardiopathy (CCC). Patients were excluded if they had not been clinically stable in the last month (i.e., deterioration and/or acute complication related or not to their preexisting condition), had comorbidities, or were not cognitively able to complete the questionnaire. Data were collected from July 2004 to June 2005.

An additional convenience sample of healthy children and adolescents was gathered to assess comprehensibility and test discriminant validity. Eligibility criteria for this sample were: (1) 2–18 years old, (2.a) attending the "Healthy Children Outpatient Clinic" at one of the three pediatric hospitals in the city or (2.b) students at one elementary school in the outskirts of Buenos Aires. These recruitment sources were selected because the socio-demographic characteristics of children were similar to those of the chronically ill children cared for at Hospital Garrahan. The study was approved by Hospital Garrahan's IRB. Parents or legal guardians granted written permission and children 10 years old and above were asked for assent.

## Instruments

### The PedsQL™ 4.0 Generic Core Scales

The 23-item PedsQL™ 4.0 Generic Core Scales encompass: 1) Physical Functioning (8 items), 2) Emotional Functioning (5 items), 3) Social Functioning (5 items), and 4) School Functioning (5 items), and were developed through focus groups, cognitive interviews, pre-testing, and field testing measurement development protocols[5,6] The instrument takes approximately 5 minutes to complete[5,6] The PedsQL™ Scales are comprised of parallel child self-report and parent proxy-report formats. Child self-report includes ages 5–7, 8–12, and 13–18 years. Parent proxy-report includes ages 2–4 (toddler), 5–7 (young child), 8–12 (child), and 13–18 (adolescent), and assesses parent's perceptions of their child's HRQOL. The items for each of the forms are essentially identical, differing in developmentally appropriate language, or first or third person tense. The instructions ask how much of a problem each item has been during the past one month. A 5-point Likert response scale is utilized across child self-report for ages 8–18 and parent proxy-report (0 = never a problem; 1 = almost never a problem; 2 = sometimes a problem; 3 = often a problem; 4 = almost always a problem). To further increase the ease of use for the young child self-report (ages 5–7), the response scale is reworded and simplified to a 3-point scale (0 = not at all a problem; 2 = sometimes a problem; 4 = a lot of a problem), with each response choice anchored to a happy to sad faces scale[28,29].

Items are reverse-scored and linearly transformed to a 0–100 scale (0 = 100, 1 = 75, 2 = 50, 3 = 25, 4 = 0), so that higher scores indicate better HRQOL. Scale Scores are computed as the sum of the items divided by the number of items answered (this accounts for missing data). If more than 50% of the items in the scale are missing, the Scale Score is not computed. This accounts for the differences in sample sizes for scales reported in the Tables. Although there are other strategies for imputing missing values, this computation is consistent with the previous PedsQL™ peer-reviewed publications, as well as other well-established HRQOL measures [6,30,31]. The Physical Health Summary Score (8 items) is the same as the Physical Functioning Scale. To create the Psychosocial Health Summary Score (15 items), the mean is computed as the sum of the items divided by the number of items answered in the Emotional, Social, and School Functioning Scales.

The adaptation of the PedsQL™ 4.0 Generic Core Scales into Argentinean Spanish was conducted following internationally accepted guidelines for cross-cultural adaptation of patient reported outcome instruments[17,32,33]. The forward translation into Spanish of all the PedsQL™ corresponding versions was conducted by two of the authors (VD, GM), a paediatrician and a child psychologist who are fluent in English. This first draft was reviewed by a multidisciplinary team that included the two authors, a child oncologist, and a health services researcher/clinician. After extensive discussion we ended up with a reconciled first Argentinean Spanish PedsQL™ version. The back translation was done by a native English speaker fluent in Spanish not familiar with the instrument. Some items were slightly modified to ensure semantic and conceptual equivalence of the second Argentinean Spanish PedsQL™ version. Cognitive debriefing interviews were carried out in two waves, first with 15 children and their parents. This pretest prompted changes that essentially involved spelling out both the main question and answer options more thoroughly (e.g. "problems with running" instead of "running" and "never was a problem" instead of "never") to increase comprehensibility. The second wave of cognitive interviews was carried out in 30 children and parents and confirmed that the final Argentinean Spanish PedsQL™ was understandable and conceptually equivalent to the original instrument. All changes and revisions were reviewed and accepted by JV.

#### Overall Health Status Ratings

**Figure 1:**
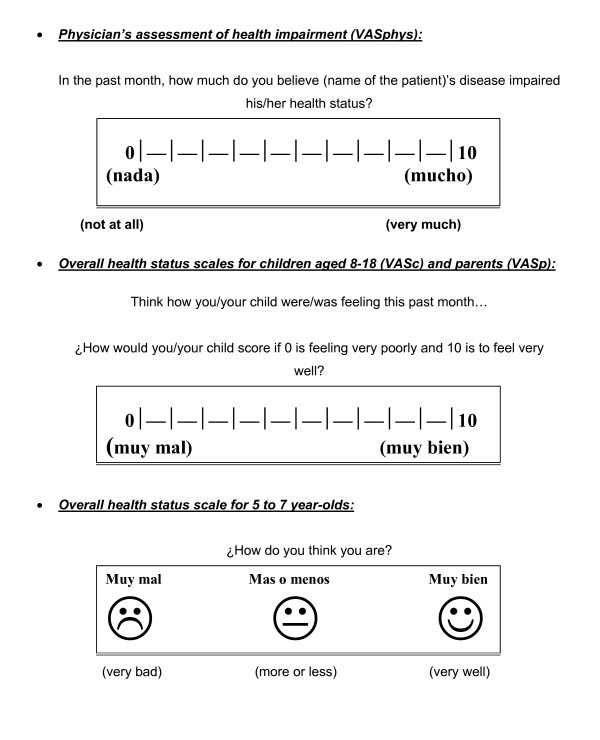
**Visual analogue scales used to measure overall health status**. Visual analogue scales (VAS) used to measure overall health status. Upper panel shows the VAS presented to physician's to assess degree of heath impairment in the past month. Middle panel shows VAS presented to older children and parents to assess overall health status in the past month. Lower panel shows faces scale used to assess self-reported overall health status in children aged 5 to 7 years old.

Overall health status ratings were developed for this study (see Figure 1). Physicians were prompted to assess the child's degree of health impairment due to their disease over the past month using a 0–10 visual analogue scale (VAS) where 0 was "no impairment at all" and 10 "maximum impairment". Children 5 years old and above and their proxies were asked to independently score how they considered the child was feeling over the last month. Children 8 years old and above and adults used a 0–10 VAS, where 0 was "very bad" and 10 "very well", whereas 5 to 7 year-olds used a three-point faces scale (very bad, more or less, very well) similar to the faces scale used in the corresponding PedsQL™ version.

#### Cognitive Debriefing/Feasibility

Children and proxy's impressions about the Argentinean Spanish version of PedsQL™, including difficulty with format and understanding, easiness, and comprehensibility were asked with a semi-structured cognitive interview.

#### Clinical and Socio-demographic variables

Clinical information such as diagnosis, disease severity, and duration of disease was abstracted from the patients' medical records and, when not available, was collected from the patients' primary physicians.

Age, gender, education level of the child and adult proxy, and socioeconomic status were collected from adult proxies. Socioeconomic status variables included health insurance (union health insurance/private insurance/disability allowances/uninsured), and poverty level, which was dichotomized as above or below the poverty line according to the ratio income/basic family living costs[34].

#### Design

This is a cross-sectional descriptive study. One interviewer (MR, not related to patient care) administered the PedsQL™ 4.0 and the validation questionnaire to all enrolled families.

Construct validity was assessed by testing the following hypothesis: (1) PedsQL™ 4.0 scores would correlate negatively with physician's assessed impairment of health status; (2) PedsQL™ 4.0 scores would correlate positively with self/proxy-reported overall health status; (3) Child self-reported and parent proxy-reported PedsQL™ 4.0 scores would correlate significantly in the medium effect size range. In addition, we used the known-groups approach to test discriminant validity by comparing PedsQL™ 4.0 scores of healthy children with those of children with chronic health conditions, as well as scores across different chronic conditions groups. It was anticipated that children with chronic health conditions would report significantly lower PedsQL™ scores overall in comparison to healthy children[19].

#### Procedures

For the field test, outpatient clinic rosters were reviewed with primary physicians who identified subjects that met inclusion criteria. Families were then approached in the clinic before seeing their doctor and invited to enroll in the study. After enrolling, children and proxies were asked to independently complete the PedsQL™ followed by the cognitive debriefing interview. Overall health status assessment was carried out after the PedsQL™ administration to avoid cuing. Proxies provided socio-demographic information at the end. Primary physicians were asked to report the child's overall health impairment after they saw the patient.

The following variables were collected by the interviewer as patients completed the instruments: (1) mode of administration (self-administered, required interviewer-administration), (2) version used (as per PedsQL™ guidelines when a patient did not understand their age-specific version they were offered the next younger age version), (3) completion time, (4) need for help (classified in 3 categories: no help, minimal help: < 4 times, and significant help: ≥ 4 times during questionnaire administration), and (5) missing items.

### Statistical Analysis

To assess the appropriateness of the PedsQL™ administration in the Argentinean public health setting we set an *a priori *condition indicating that at least 80% of the questionnaires should be answered based on an empirical consideration that if more than 20% of the targeted sample was not able to complete the questionnaire, the tool would not serve the purpose of generating valid, representative data[35]. Questionnaires were considered unanswered if they took more than 30 minutes to complete (this was considered a reasonable time for research purposes given that not everyone was expected to take so long) or if more than 50% of items were not understood despite interviewer's assistance (following the author's guidelines[36] of not scoring questionnaires with more than 50% of missing items[31]). In addition, the association between comprehensibility and sociodemographic covariates was analyzed using T-test for independent samples and Chi Square or Fisher's exact test as appropriate. A p-value < 0.05 was considered significant.

Descriptive statistics of the items, average scores, as well as ceiling and floor effects are reported. Ceiling and floor effects were considered present if > 15% of respondents used the extreme values[37]. Scores were stratified by respondent, age group, and type of chronic condition. Scale reliability was evaluated using Cronbach's coefficient alpha. Construct validity was tested using Pearson's correlation coefficient. Discriminant validity was evaluated by testing differences among chronic and healthy children scores, disease subgroups, gender, and SES using t-test or ANOVA for binary and categorical variables respectively. Data analysis was conducted with SPSS 10.0 for Windows.

## Results

**Figure 2:**
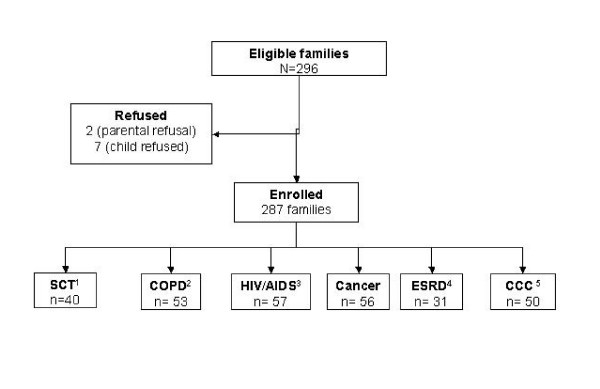
**Flowchart and Patient Diagnosis for the Argentinean Spanish Validation of PedsQL™ 4.0 in children with chronic conditions**. ^1^Hematopoietic Stem Cell Transplant ^2^Chronic Obstructive Pulmonary Disease ^3^Human Immunodeficiency Virus infection or Acquired Immune Deficiency Syndrome ^4^End Stage Renal Disease ^5^Complex Congenital Cardiopathies.

Among 296 eligible families of children with chronic conditions 287 (96%) enrolled. Figure 2 presents the study flowchart and diagnosis of the enrolled families. In Table 1 their clinical and socio-demographic characteristics are presented.

**Table 1:** Characteristics of children with chronic health conditions. Argentinean Spanish Validation of the PedsQL™ 4.0 Generic Core Scales.

	**Age Group**				
		
	**2–4** ** years old** ** n = 70**	**5–7** ** years old** ** n = 62**	**8–12** ** years old** ** n = 90**	**13–18** ** years old** ** n = 65**	**TOTAL** ** N = 287**
Patient gender					
Female	41.5%	39%	42%	46%	42%
Male	58.5%	61%	58%	54%	58%
Proxy respondent					
Mother	80%	76%	70%	61.5%	72%
Father	14%	14.5%	20%	9.5%	15%
Other	6%	9.5%	10%	29%	13%

**Chronic condition**					
SCT^1^(n = 40)	7%	8%	16%	25%	14%
COPD^2 ^(n = 53)	24%	29%	14%	8%	18%
HIV/AIDS^3^(n = 57)	19%	24%	19%	18%	20%
Cancer (n = 56)	21%	20%	18%	20%	20%
ESRD^4^(n = 31)	3%	3%	18%	17%	11%
CCC^5^(n = 50)	26%	16%	15%	12%	17%
Time since diagnosis in months, median (range)	28 (1–60)	63 (1–84)	89 (1–148)	95 (2–204)	48 (1–204)

**Socio-Demographics**					
Education					
Child below appropriate for age	-------	11%	29%	34%	25%
Proxy did not complete elementary school	3%	11%	4.5%	15.5%	11%

Below the poverty line^6^	67%	71%	65%	60%	66%
No health Insurance	67%	61%	44.5%	46%	54%

^1^Stem Cell Transplant ^2^Chronic Obstructive Pulmonary Disease ^3^Human Immunodeficiency Virus infection or Acquired Immune Deficiency Syndrome ^4^End Stage Renal Disease ^5^Complex Congenital Cardiopathies ^6^Poverty line is calculated according to total income, and number and age of people in the household, *as per *National Institute of Statistics and Census (INDEC) guidelines.

The distribution of socio-demographic characteristics across the different age groups was homogenous, with slight predominance of males in all of them. Twenty-five percent of children were below the appropriate school level for their age, and 6% were not attending school; 11% of adult respondents had not completed elementary school and 3.2 % were functional illiterates. Most surveyed families lived below the poverty line (66%) and 54% had no health insurance.

Out of 107 eligible families of healthy children, 105 (98%) enrolled. Healthy children were comparable to those with chronic conditions, except for gender and socioeconomic status. Healthy children were more likely to be females (55% vs. 42%, p = 0.023), have no medical insurance (74% vs. 54%, p = 0.001), and less likely to live below the poverty line (54% vs. 66%, p = 0.046).

### Feasibility

In Table 2, we present feasibility of administering the Argentinean Spanish version of the PedsQL™ 4.0. Overall, the instrument was well understood. Median time to completion was 6 minutes for children (range 2–28') and 5 minutes for adults (range 1–16'). In 54% of the cases the age-appropriate questionnaire was completed without help and in 27.5% with minimal help. The need for help decreased with age. Among the 217 children with chronic conditions surveyed, 9 (4.1%), all aged 5 to 7 years, were not able to understand and complete the questionnaire and 7 (3.5%), all aged 8 to 12 years, needed to use the young child version for 5–7 year olds. An additional 7%, mostly 8–12 year-olds, required the PedsQL™ to be administered by the interviewer. No health condition was associated with not being able to answer. No adult questionnaire was unanswered. Main difficulty for adults was with format, 12.5% forgot to complete an item or more and needed to be prompted by the interviewer in order to complete it adequately.

**Table 2:** PedsQL 4.0 Argentinean Spanish Administration: Difficulties, help, and time to completion in children with chronic conditions

	**Children **				**Adults**
	
	**5–7 yo** ** n = 62^1^**	**8–12 yo** ** n = 90**	**13–18 yo** ** n = 65**	**TOTAL** ** n = 217**	**Total** ** n = 287**
**Time to completion, minutes**					
Median (range)	5'(3–20)	7'(2–28)	5'(2–12)	6'(2–28)	5'(1–16)
**Required Help^1^**					
No	42%	46.5%	77%	54.5%	69%
Minimal	32%	29%	21.5%	27.5%	26%
Significant	11.5%	24.5%	1.5%	14%	5%
**Form of Administration**					
Adequate	100%	78%	97%	89,5%	95,5%
Administered by interviewer	N/A^2^	14.5%	3%	7%	4,5%
Previous version	N/A^3^	7.5%	0%	3.5%	N/A^4^
**Difficulties with the format**					
Forgot	N/A^2^	10%	3%	5.5%	12.5%
Wrote over other item	N/A^2^	11%	4.5%	6.5%	4%

^1^Minimal help: < 4 times, Significant: ≥ 4 times during questionnaire administration.

^2^N/A: not applicable, always administered by interviewer

^3^N/A: not applicable, No existence of previous versions

Poverty and a low education level were significantly associated with requiring more help to complete the PedsQL™ 4.0 for both children and parents (Table 3). When both poverty and low education level were present, 30% of children and 19% of parents required significant help whereas only 15% of children and 4% of parents required significant help if they were not in this category (p = 0.049 for children and 0.001 for parents). All but one of the children who could not complete the questionnaire lived below the poverty line.

**Table 3:** PedsQL 4.0 Argentinean Spanish Administration: Socioeconomic status, education and requirement of help to complete PedsQL in children with chronic conditions and their parents

	**Required Help^1^**			
	
	**No help**	**Minimal **	**Significant **	p-value^2^
**Living below poverty line**				
Children	69 (47%)	41 (29%)	32 (23%)	0.025
Parents	122 (65%)	53(28%)	14 (7%)	0.026
**Low education**				
Children (lower than expected)	22 (40%)	16 (29%)	17 (31%)	0.008
Parents (incomplete elementary school)	17 (53%)	10 (31%)	5 (16%)	0.030
**Low income and low education**				
Children	18 (42%)	12 (28%)	13 (30%)	0.049
Parents	12 (44%)	10 (37%)	5 (19%)	0.001

^1^Minimal help: < 4 times, Significant: ≥ 4 times during questionnaire administration or could not complete questionnaire. ^2^Chi-square test

There were few missing items. Children only left 2.4% (115/4784) items unanswered whereas adults left 4.3% (218/6461). Five items from the school dimension were responsible for 78% of children's and 90% of adult's missing items, and corresponded to children that were not going to school.

Almost all children (95%) and parents (96%) considered the questions relevant, a large proportion found them easy to answer (81% of children and 91% of parents), and most said the paper format was friendly (91% of children and 98% of parents).

### Scores Distribution

In Table 4, average summary and scales scores, standard deviations and range, as well as ceiling and floor effects are presented. Children and adults used the complete range of response options for all 23 items with a slight deviation towards the uppermost end. Ceiling and floor effects were negligible for all dimensions but the social domain, where a moderate ceiling effect (20.2%) was observed in proxy respondents.

**Table 4:** Scale Descriptives for Argentinean Spanish version of the PedsQL 4.0 Generic Core Scales Child Self-Report and Proxy-Report

**Scale**	**Scale Descriptives**					
	
	**Mean ± SD^1^**	**Range**	**Floor Effect^2^** **(%)**	**Ceiling Effect^2 ^** **(%)**	**N**	**α^3^**
**Self-Report**						

Total	66.87 ± 16.74	26–99	0	0	177	0.86
Physical	67.76 ± 19.6	0–100	0.5	4.8	196	0.69
Psychosocial	66.36 ± 17.49	27–100	0	0.5	186	0.80
Emotional	65 ± 21.31	0–100	0.5	5.3	208	0.59
Social	69.1 ± 21.67	10–100	0	11.1	203	0.59
School	65.6 ± 21.3	10–100	0	5.2	189	0.62

**Proxy-Report**						

Total	73.36 ± 16.09	14–100	0	1.7	183	0.87
Physical	74.67 ± 20.06	4–100	0	10.1	272	0.78
Psychosocial	72.41 ± 16.45	18–100	0	2.4	189	0.81
Emotional	69.16 ± 19.6	5–100	0	6.3	285	0.66
Social	77.78 ± 20.73	5–100	0	20.2	283	0.71
School	68.74 ± 24	5–100	0	1.7	192	0.68

^1 ^Higher mean values indicate better HRQOL (range 0–100).

^2^Floor and ceiling effects are considered present if > 15% of extreme values were used

^3^Cronbach α Coefficient.

Older children had significantly higher scores than younger children (Table 5), except for the emotional dimension. In contrast, parent proxy-report scores for the 2–4 year-olds were significantly higher than proxy-report scores of older children.

**Table 5:** PedsQL 4.0 Argentinean Spanish Scores and internal consistency by age group. (Analysis of Variance – ANOVA)

	**2–4 yo**			**5–7 yo**			**8–12 yo**			**13–18 yo**			
**Score/Dimension**	**N**	**Mean** **Total** **Score** ** (SD)**	**α^1^**	**N**	**Mean** **Total** **Score** ** (SD)**	**α^1^**	**N**	**Mean** **Total** **Score** ** (SD)**	**α^1^**	**N**	**Mean ** **Total ** **Score** ** (SD)**	**α^1^**	**Differences^2^**

**Self-Report **													

Total	N/A	N/A^3^	N/A	43	60.03 (15.86)	0.76	76	66.75 (16.8)	0.86	58	72.6 (15.41)	0.89	
Physical	N/A	N/A	N/A	49	62.20 (20.40)	0.57	85	66.36 (20.24)	0.73	72	74.24 (16.24)	0.71	13–18yo > 5–7yo*** 13–18yo > 8–12yo*
Psychosocial	N/A	N/A	N/A	45	58.81 (16.13)	0.65	80	66.98 (17.34)	0.81	61	71.67 (16.83)	0.86	8–12yo > 5–7yo** 13–18yo > 5–7yo***
Emotional	N/A	N/A	N/A	53	62.07 (23.56)	0.45	90	63.47 (21.03)	0.62	65	69.54 (19.3)	0.72	NS
Social	N/A	N/A	N/A	49	57.23 (20.64)	0.28	89	70.05 (20.68)	0.59	65	77.61 (19.64)	0.73	8–12,13–18yo > 5–7yo***
School	N/A	N/A	N/A	47	56.80 (20.86)	0.44	81	68.47 (21.69)	0.65	61	68.47 (19.53)	0.69	8–12yo > 5–7yo*** 13–18yo > 5–7yo**

**Proxy-Report**													

Total	66	80.15 (13.19)	0.62^4^	50	73.88 (16.26)	0.89	80	69 (21.24)	0.84	53	71.25 (17.18)	0.89	2–4yo > 8–12,13–18yo***
Physical	66	82.34 (14.94)	0.65	58	74.78 (21.3)	0.86	87	70.12 (21.24)	0.77	61	72.59 (20)	0.78	2–4yo > 8–12*** 2–4yo > 13–18yo*
Psychosocial	69	78.41 (14.29)	0.30^5^	53	73.33 (16.5)	0.83	81	68.4 (15.62)	0.77	55	70.62 (18.01)	0.84	2–4yo > 8–12*** 2–4yo > 13–18yo*
Emotional	70	75.46 (15.4)	0.54	62	69.68 (19.97)	0.73	90	66.05 (19.04)	0.62	63	66.17 (22.58)	0.75	2–4yo > 8–12,13–18yo*
Social	69	83.86 (17.65)	0.65	61	77.38 (20.10)	0.65	90	73.67 (21.95)	0.72	63	77.30 (21.54)	0.79	2–4yo > 8–12yo*
School	20	73.68 (13.96)	0.47	54	71.76 (22.06)	0.74	81	65.72 (22.4)	0.65	57	68.65 (20.55)	0.64	NS

^1 ^Cronbach α Coefficient ^2^p values based on analysis of variance (ANOVA) comparing the mean scores across age groups *p < .05 **p < 0.01 ***p < .005 with Bonferroni correction for the number of comparisons, p < .005 values should be considered statistically significant. NS: non significant, p > 0.05. ^3^N/A: not applicable ^4^If school items are excluded, α = 0.83. ^5 ^If school items are excluded, α = 0.76.

### Reliability

Cronbach's alpha coefficients for the summary and scale scores for all children with chronic conditions are presented in Table 4. Table 5 presents results by age group. The internal consistency of the total scores, and the physical and psychosocial subscale scores exceeded the 0.70 minimum usually accepted for group comparison for all age groups except for the 2–4 year-olds proxy-report, and the physical functioning and psychosocial subscales of the 5–7 year-olds self-report (α = 0.57 and α = 0.65 respectively). In the 2–4 year-old group, educational items were missing for 51 (72.9%) patients. When these three items were excluded, internal consistency increased markedly (total α = 0.83 and psychosocial α = 0.76)). Emotional, social, and school subscales had overall lower reliability although the proxy-reports of the 5–7, 8–12, and 13–18 year-olds, and the 13–18 year-olds self-report were close or superior to the 0.70 mark (except for the emotional subscale of the 8–12 year-old proxy-reports with an α = 0.62) and below 0.65 for the other groups (the 5–7 year-old self-reports being the lowest). Among child self-reports, internal consistency increased with age.

### Construct Validity

As hypothesized, there was a significant and negative correlation between the primary physician's assessment of health impairment status (VASphys) and both self-report and proxy total PedsQL™ 4.0 scores (Table 6). Correlation between total PedsQL™ scores and overall self-reported/proxy health status evaluation was significant and positive in both children and adults. Total self-report and proxy-report scores were also significantly correlated. Of note, self-report global scores were significantly lower than proxy-report global scores. All correlations were in the moderate range (<> 0.20–0.50).

**Table 6:** Construct validity of the PedsQL 4.0 Argentinean Spanish Version

**1^st ^Hypothesis: Correlation between PedsQL scores and overall health impairment^1^**				
	**PedsQL** ** mean (SD)**	**Physician VAS** ** mean (SD)**	**r^3^**	**p-value**
Self-Report	66.87 (16.74)	4.01(2.42)	-0.23	0.001
Proxy-Report	73.36 (16.09)	4.01(2.42)	-0.32	< 0.001

**2nd Hypothesis: Correlation between PedsQL scores and self-reported overall health status^2^**				
	**PedsQL** ** mean (SD)**	**Self-report VAS** ** mean (SD)**	**r^3^**	**p-value**
Self-Report	66.87 (16.74)	8.32 (1.82)	0.34	< 0.001
Proxy-Report	73.36(16.09)	8.38 (1.51)	0.33	< 0.001

**3^rd ^Hypothesis: Correlation between Self-report and Proxy-report PedsQL scores^2^**				
	**Self-Report**	**Proxy-Report**	**R^3^**	**p-value**
PedsQL, mean (SD)	66.87 (16.74)	73.36 (16.09)	0.39	< 0.001

^1^VASphys: 0 (none) to 10 (maximum) impairment of health status

^2^VASc or VASp: 0 (very bad) to 10 (very well) overall feeling during the past month.

^3^r = Pearson's correlation coefficient interpreted as low (< 0.10), moderate (0.11–0.30) and high (> 0.30).

### Discriminant Validity

As expected, child self-report and parent proxy-report total, physical, and psychosocial scores for healthy children were on average significantly higher than those of children with chronic conditions (Table 7) except for the emotional and school self-report subscales. PedsQL™ 4.0 total scores also varied significantly across health conditions for both self-reports and proxy-reports (Table 7). Patients with COPD, ESRD, or cancer reported the lowest scores.

**Table 7:** Comparison of PedsQL 4.0 Argentinean Spanish scores of healthy children and children with chronic conditions

	**Healthy children**	**All Chronic conditions**	**p-value^1^**	**Scores by illness group**						
				
				**SCT^2^** **Mean (SD)**	**COPD^3^** **Mean (SD)**	**HIV/AIDS^4 ^** **Mean (SD)**	**Cancer** ** Mean (SD)**	**ESRD^5^** **Mean (SD)**	**CCC^6^** **Mean (SD)**	**Differences^7^**
**Self-Report **										

Total	72.72 (14.21)	66.87 (16.74)	0.011	71.64 (17.13)	58.54 (15.8)	71.31 (17.07)	65.16 (15.45)	65.29 (19.96)	68.98 (15.22)	SCT, HIV > COPD*
Physical	75.42 (15.93)	67.76 (19.60)	0.004	74.24 (17.08)	58.16 (19.84)	74.58 (18.05)	60.98 (19.44)	67.17 (1.32)	72.03 (15.84)	SCT, HIV > Cancer* SCT > COPD** HIV > COPD***
Psychosocial	71.20 (14.84)	66.36 (17.49)	0.028	70.10 (18.51)	58.76 (16.43)	69.52 (17.69)	67.57 (6.46)	64.07 (7.45)	67.32 (7.04)	NS^8^

Proxy-Report*										
Total	82.19 (12.97)	73.36 (16.09)	< 0.001	75.46 (15.99)	69.61 (18.29)	79.32 (13.3)	71.39 (14.79)	66.92 (18.8)	74.61 (13.52)	HIV > COPD, ESRD*
Physical	86.20 (12.27)	74.67 (20.06)	< 0.001	77.13 (17.44)	67.85 (23)	83.98 (14.23)	69.92 (18.82)	66.83 (26.28)	79.49 (15.78)	HIV > COPD, Cancer, ESRD*** CCC > COPD*
Psychosocial	79.91 (14.96)	72.41 (16.45)	< 0.001	74.46 (17.89)	70.38 (18.47)	76.74 (14.77)	72.24 (16.34)	66.93 (16.53)	71.57 (14.02)	NS^7^

^1 ^Student's t test ^2^Stem Cell Transplant ^3^Chronic Obstructive Pulmonary Disease ^4^Human Immunodeficiency Virus infection or Acquired Immune Deficiency Syndrome ^5^End Stage Renal Disease ^6^Complex Congenital Cardiopathies ^7^p values based on analysis of variance (ANOVA) * p < .05 **p < .01 ***p < .005 With a Bonferroni correction for the number of comparisons, p < 0.005 should be considered statistically significant. Higher values equal better health-related quality of life. ^8^NS: non-significant

Children living below the poverty line were more likely to have lower total PedsQL™ scores (65.38 vs 70.29 respectively, p = 0.035) than their counterparts. These were mainly due to significantly lower emotional and school functioning scores. No statistically significant differences were found between PedsQL™ scores and gender.

### Comparison with other cross-cultural adaptations

Table 8 presents how results from our study compare to the original validation study and other published cross-cultural validations of PedsQL™. For most cross-cultural validation studies population characteristics differed from ours. Target population was commonly restricted to school children, and thus children were older and healthier. In addition, because of country characteristics, socioeconomic status tended to be higher compared to the Argentinean families we recruited. Our scores were overall lower than most of the other validation studies, including those that included similar age ranges and conditions. Reliability was reported in different ways across these studies, but the lower bound of internal consistencies found by our study was lower than the ones reported for most of the other validation studies. Types of validity tested and findings were similar to those reported by the other cross-cultural adaptations.

**Table 8:** PedsQL 4.0 cross-cultural adaptation's reliability and validity. Comparison of published studies.

				**Scores**							
						
**Study**	**Sample characteristics**	**N**	**Age groups**	**Self-Report**			**Proxy Report**			**Reliability range**	**Type of Validity tested**
						
				Total	Physical	Psychosocial	Total	Physical	Psychosocial		
**Our Study**	Healthy Children	105	2–18 yo	72.72	75.42	71.20	82.19	86.20	79.91		Known groups validity Convergent validity Self-report/Proxy correlations
	Chronic conditions	287	2–18 yo	66.87	67.76	66.36	73.36	74.67	72.41		
**US Original^1^‡**	Well-child visits, clinic visits, children who had an admission	1645	2–18 yo	79.62	80.19	79.37	80.87	81.38	80.58	0.68–0.90	Known groups validity Predictive validity Factor analysis
**Austria^2^**	School children	1412	8–12 yo	81.9	87.8	79.9	84.9	90.6	83.1	NR	Construct validity Predictive validity
**Finland^3^**	School children	1097	8–12 yo	81.54	85.57	78.68	77.61	79.20	76.26	0.69–0.91	Compared to US study results
**Germany^4^**	Chronic conditions	41 (epilepsy)	2–17 yo	78.0	87.3	NR	76.7	84.1	NR	0.72–0.91	Known groups validity Self-report/Proxy correlations
		126 (cancer)		82.6	86.7	NR	80.4	85.0	NR	0.60–0.84	
**Greece^5^**	School children	645	8–12 yo	82.10	84.27	80.94	83.11	87.75	80.67	0.65–0.84	Factor analysis Self-report/Proxy correlations
**Iceland^6^**	School children	480	10–12 yo	Not reported summarized						NR	Predictive validity Known groups validity
**Norway^7^**	School children	425	13–15 yo	85.29	91.12	82.16	86.10	88.83	84.66	0.73–0.88	Factor analysis Convergent correlation Self-report/Proxy correlations
**UK^8^**	School children	1399	2–18 yo	83.89	88.51	81.84	84.61	89.06	82.21	> 0.70	Known groups validity Self-report/Proxy correlations
	Chronic conditions	365	2–18 yo	Scores were reported for each condition but not summarized							
**Turkey^9^**	Healthy children, children with acute and chronic conditions	223	2–4 yo	NA	NA	NA	78.17	79.40	77.25	0.66–0.85	Known groups validity Self-report/Proxy correlations
		198	5–7 yo	71.56	72.66	70.82	72.92	69.96	74.76	0.57–0.86	
**Japan^10^**	School children	229	6–13	76.7	83.4	73.3	81.4	92.6	75.8	0.71–0.86	Known groups validity Self-report/Proxy correlations Factor analysis
	Chronic conditions	100	5–18	NR	NR	NR	NR	NR	NR	NR	
**Catalunya^11^**	School children	511	9–17 yo	81.53	88.26	79.23	-	-	-	0.76–0.80	Known groups Convergent validity (compared with KINDL scores) Predictive validity

‡ PedsQL 4.0 has undergone multiple validation studies in the US. A summary of the results and citations is provided in the introduction.

NR: Not reported

1. Varni JW, Seid M, Kurtin PS. PedsQL 4.0: reliability and validity of the Pediatric Quality of Life Inventory version 4.0 generic core scales in healthy and patient populations. *Med Care *2001;**39**(8)**:**800–12.

2. Felder-Puig R, Baumgartner M, Topf R, Gadner H, Formann AK. Health-Related Quality of Life in Austrian Elementary School Children. *Med Care *2008;**46**(4)**:**432–439.

3. Laaksonen C, Aromaa M, Heinonen OJ, Suominen S, Salantera S. Paediatric health-related quality of life instrument for primary school children: cross-cultural validation. *J Adv Nurs *2007;**59**(5)**:**542–50.

4. Felder-Puig R, Frey E, Proksch K, Varni JW, Gadner H, Topf R. Validation of the German version of the Pediatric Quality of Life Inventory (PedsQL) in childhood cancer patients off treatment and children with epilepsy. *Qual Life Res *2004;**13**(1)**:**223–34.

5. Gkoltsiou K, Dimitrakaki C, Tzavara C, Papaevangelou V, Varni JW, Tountas Y. Measuring health-related quality of life in Greek children: psychometric properties of the Greek version of the Pediatric Quality of Life Inventory(TM) 4.0 Generic Core Scales. *Qual Life Res *2008;**17**(2)**:**299–305.

6. Svavarsdottir EK, Orlygsdottir B. Health-related quality of life in Icelandic school children. *Scand J Caring Sci *2006;**20**(2)**:**209–15.

7. Reinfjell T, Diseth TH, Veenstra M, Vikan A. Measuring health-related quality of life in young adolescents: reliability and validity in the Norwegian version of the Pediatric Quality of Life Inventory 4.0 (PedsQL) generic core scales. *Health Qual Life Outcomes *2006;**4:**61.

8. Upton P, Eiser C, Cheung I, et al. Measurement properties of the UK-English version of the Pediatric Quality of Life Inventory 4.0 (PedsQL) generic core scales. *Health Qual Life Outcomes *2005;**3:**22.

9. Uneri OS, Agaoglu B, Coskun A, Memik NC. Validity and reliability of Pediatric Quality of Life Inventory for 2- to 4-year-old and 5- to 7-year-old Turkish children. *Qual Life Res *2008;**17**(2)**:**307–15.

10. Chen X, Origasa H, Ichida F, Kamibeppu K, Varni JW. Reliability and validity of the Pediatric Quality of Life Inventory (PedsQL) Short Form 15 Generic Core Scales in Japan. *Qual Life Res *2007;**16**(7)**:**1239–49.

11. Huguet A, Miro J. Development and psychometric evaluation of a Catalan self- and interviewer-administered version of the Pediatric Quality of Life Inventory version 4.0. *J Pediatr Psychol *2008;**33**(1)**:**63–79.

## Discussion

Our study results provide initial evidence towards the reliability and validity of the Argentinean Spanish version of the PedsQL™ 4.0 Generic Core Scales in the public health research setting. The Argentinean Spanish version of the PedsQL™ 4.0 has good feasibility. It was easy to administer, completed without or with minimum help by most children and parents, required a short administration time (not more than 5–6 minutes on average), and only 4.1% of children (all 5–7 year-olds) could not complete the instrument. However, our results suggest that some sort of help, albeit small, is needed for many, especially for children and parents from lower socioeconomic strata and low literacy levels. Internal consistency approached or exceeded that required for group comparisons for children over 8 years old and parents of children over 5 years old. The Argentinean Spanish version of the PedsQL™ 4.0 showed good construct and discriminant validity properties in this low-income setting, making this instrument suitable for research use. In order to expand the use of the PedsQL™ 4.0 in Argentinean children, an alternative approach to scoring for the 2–4 year-olds should be considered along with further understanding of how to increase reliability for the 5–7 year-old self-report and assessment of other instrument characteristics such as responsiveness and sensitivity to change.

Our initial concern that socioeconomic status and literacy may influence people's ability to use PedsQL™ 4.0 seems to be supported by our data, although to a lesser extent than was expected. As a matter of fact, all children that could not complete PedsQL™ 4.0 lived below the poverty line and both children and parents who were poor and had low literacy levels were more likely to require help with the instrument. Nevertheless, the 14.5% of 5–7 year-olds who could not complete PedsQL™ was lower than the 38% observed in the German validation of the PedsQL™[38], and was also within our a priori requirement of < 20% unanswered questionnaires. Importantly, all the parents were able to complete the questionnaire, albeit with assistance, even those that had not completed elementary school or were functional illiterates. The main implications of these findings are that in order to use PedsQL™ in our public health setting, availability of trained interviewers during questionnaire administration needs to be assured, especially for children and parents who are poor and have low literacy levels. In addition, carefully thought training guidelines for children and parents should be developed and tested.

The Argentinean Spanish PedsQL™ version had lower reliability compared to other validation studies[11-13,15,16,18,20,38,39]. Given the low prevalence of school attendance among the 2–4 year olds with chronic conditions, this version of the Argentinean Spanish PedsQL™ may work better if school items are not taken into consideration for scoring purposes in this group. In addition, although Cronbach alpha represents the lower bound of the reliability of a measurement instrument, and is a conservative estimate of actual reliability[40], scales that did not approach or meet the 0.70 standard should be used only for descriptive analyses. Self-report scores of 5–7 year-olds presented the lowest internal consistency values. Of note, these children had the most difficulty with completing PedsQL™, which may be indicating that results of the Argentinean Spanish PedsQL™ version for this age group may not be as reliable as for the older groups. Although these results are somewhat comparable to the German validation[38], other studies in this age group [9,20] have showed higher alpha coefficients and less problem with instrument completion. HRQOL measurement in young children is still challenging and our results warrant further research including larger samples[41,42].

Construct validity was assessed in a similar fashion to other validation studies[6,12-14,16,20,38,43] and supported by our data. The self-reported health status VAS scales had not been used before in our setting, but there is substantial evidence that VAS scales are reliable and valid tools to assess general health status [44]. Of note, all correlations were in the moderate range which indicates that although statistically significant they are not highly predictive of one another.

Our results also indicate that the Argentinean Spanish version of the PedsQL™ 4.0 has good discriminant validity. The Argentinean Spanish version of the PedsQL™ was able to distinguish between healthy and chronically ill children and between those with different chronic health conditions, as previously reported for the U.S. English version[19]. As was found in previous studies[8,11], the Argentinean Spanish PedsQL™ was also able to discriminate between SES levels. Interestingly, the Total Scale Score and scale scores of the Argentinean Spanish version of the PedsQL™ were consistently lower than those reported in the original publication[6] and almost all published cross-cultural adaptations[11-16,18,38,39,45] for both the chronically ill and healthy samples. Our results could be reflecting the socioeconomic characteristics of our sample. Compared to the general population, our sample was poorer. National statistics for Argentina [34] indicate that 46% of children ages 0–13 and 40% of children 13 and older live below the poverty line, which is lower than the 66% found in our sample. Even more, our healthy sample was purposely selected from sources that assured a higher prevalence of poverty, and in fact these children were more likely to be poorer than the general population although significantly less poor than our ill children sample. Varni et al[8], in a recent population study in schools found that Hispanics, compared to white and other ethnic origins, and those with lower SES, compared to higher SES, reported overall significantly lower PedsQL™ scores. Thus, the lower quality of life reported by the families interviewed in our study may be reflecting a combination of cultural (Hispanic culture may be associated with reports of lower quality of life independent of socioeconomic reasons) and socioeconomic determinants. To corroborate our hypothesis, future studies should include people from higher SE strata and results should then be compared locally and internationally.

### Strengths and Limitations

Our study provides innovative data regarding the use of a HRQOL instrument in the Argentinean public health setting. Our very high enrollment rate (> 90%) seems to indicate that the sample would be representative of the study base population. Further, we took special interest in trying to unveil potential difficulties in PedsQL™ use as we worried that our population's lower socioeconomic status and literacy would impair their ability to use such an instrument. Reports of the impact of lower socioeconomic status and literacy on pediatric HRQOL are not common despite its argued value[46]. Our results are encouraging and show that research on quality of life topics is not only possible in low socioeconomic settings but also relevant: surveyed families showed great enthusiasm about our paying attention to aspects of their lives that seem to be neglected frequently.

One of the main limitations of our study is that our sample size does not allow us to conduct thorough evaluations across illnesses and age groups. In addition, two important features of patient reported outcome instruments, test-retest reliability and sensitivity to change, were not assessed and are warranted to fully understand the applicability of PedsQL™ 4.0 in Argentinean children. However, generic instruments are better suited to compare across conditions than to assess specific interventions for a given condition[47] and in this context, responsiveness and sensitivity to change may be less relevant characteristics. Validation of specific HRQOL modules or instruments may be more appropriate to evaluate such changes[48]. Finally, it is also important to bear in mind that instrument validity is a concept that builds upon repeated instrument use[49].

## Conclusion

Overall, the Argentinean Spanish version of the PedsQL™ 4.0 Generic Core Scales version proved to be understandable and feasible to use. It showed good reliability for children over 8 years old and parents of children over 5 years old and good construct and discriminant validity properties in this low-income setting, making this instrument suitable for research use. Steps to expand the use of this tool should include an alternative approach to scoring for the 2–4 year-olds, further understanding of how to increase reliability for the 5–7 year-old self-report, and confirmation of other aspects of validity. Having a HRQOL instrument with demonstrated reliability and validity in the Argentinean culture will allow us to start addressing the impact of chronic illness on the quality of life of children and adolescents, including those in poor districts.

## Abbreviations

HRQOL: Health-related quality of life; PedsQL™: Pediatric Quality of Life Inventory™; VAS: Visual Analogue Scale; SCT: Allogenic hematopoietic stem cell transplantation; COPD: Chronic Obstructive Pulmonary Disease with indication of home oxigenotherapy; ESRD: End Stage Renal Disease; CCC: Complex Congenital Cardiopathies.

## Competing interests

Dr. Varni holds the copyright and the trademark for the PedsQL™ and receives financial compensation from the Mapi Research Trust, which is a nonprofit research institute that charges distribution fees to for-profit companies that use the Pediatric Quality of Life Inventory™. The PedsQL™ is available at the PedsQL™ Website[50]. The rest of the authors declare that they have no competing interests.

## Authors' contributions

All authors collaborated in the study design, MR collected the data, MR, SR, GB, and VD conducted the analysis and drafted the paper, and all authors reviewed and approved the manuscript.
